# FiberWire as a Non-Metallic Binding Material for Dorsal K-Wire Spinal Stapling: An Ex Vivo Rabbit Thoracolumbar Biomechanical Study

**DOI:** 10.3390/vetsci13101010

**Published:** 2026-09-24

**Authors:** Wonhyung Choi, Byoungho An, Gonhyung Kim

**Affiliations:** Department of Veterinary Surgery, College of Veterinary Medicine, Chungbuk National University, Cheongju 28644, Republic of Korea; 05cirdle@gmail.com (W.C.); qudgh0306@naver.com (B.A.)

**Keywords:** FiberWire, stainless steel wire, spinal stapling, vertebral fracture, vertebral luxation

## Abstract

Dorsal spinal stapling secures a Kirschner wire (K-wire) staple to adjacent spinous processes, but whether flexible non-metallic suture can replace stainless steel wire remains unclear. This study compared FiberWire and stainless steel wire in an ex vivo thoracolumbar model from 20 male New Zealand White rabbit cadavers. An isolated bone-hole tensile test was performed at L5 and L6. For constructs, a U-shaped 1.0 mm K-wire staple was passed through the L3 spinous process, positioned along both sides of T13–L2, and secured with either No. 2 FiberWire or 0.76 mm stainless steel wire. Gross flexion–extension and lateral range of motion (ROM) were measured, followed by destructive three-point bending under ventral and lateral loading. Isolated bone-hole maximum load did not differ significantly between FiberWire and stainless steel wire (89.0 vs. 118.2 N; *p* = 0.20). Both constructs reduced ROM vs. intact controls (*p* < 0.01), without differences between binding materials. Under ventral bending, FiberWire had a higher median maximum load than stainless steel wire (77.4 vs. 50.0 N; *p* < 0.05), whereas lateral bending loads did not differ. FiberWire therefore provided gross motion restriction comparable to stainless steel wire and greater resistance to ventral destructive bending, while isolated bone-hole strength did not differ significantly between materials.

## Simple Summary

Dorsal spinal stapling is a technique in which a bent metal pin is attached to several vertebral spinous processes to restrict excessive spinal movement. Stainless steel wire is commonly used to secure the pin, but its rigidity may concentrate force at the thin bone surrounding the fixation holes. This study evaluated whether FiberWire, a strong flexible surgical suture, could be used instead of stainless steel wire. Rabbit spinal specimens were tested both at individual bone holes and as complete multi-vertebral constructs. FiberWire did not increase the strength of an individual bone hole, and both materials produced similar restriction of spinal movement. However, constructs secured with FiberWire tolerated a greater load during forward bending, whereas no difference was found during sideways bending. These findings indicate that FiberWire and stainless steel wire behave differently depending on how force is applied to the complete construct. FiberWire may be considered as a non-metallic binding material for dorsal spinal stapling, although its clinical relevance remains uncertain, and further studies are needed to evaluate durability and performance in unstable spinal injuries.

## 1. Introduction

Vertebral fractures and luxations are important causes of pain, spinal instability, and neurological dysfunction in dogs and cats [1,2,3]. Surgical stabilization is usually considered when vertebral instability, progressive neurological deficits, or marked displacement are present [4,5]. Several fixation methods have been described, including pins or screws with polymethylmethacrylate, vertebral body plating, locking plate fixation, and external skeletal fixation. These techniques can provide strong stabilization because they engage the vertebral body or adjacent vertebral segments directly, but they may be technically demanding and invasive, particularly in small dogs and cats [6,7,8,9].

Spinous process-based tension band stabilization, often described as spinal stapling, has been used as a dorsal stabilization technique in selected small animals with thoracolumbar vertebral injuries. In this technique, a staple-like Kirschner wire (K-wire) or pin construct is secured to adjacent spinous processes using a binding material, commonly stainless steel wire. The construct restricts gross intervertebral motion through a dorsal tension band mechanism rather than by directly fixing the vertebral body. Therefore, spinal stapling should not be interpreted as equivalent to rigid vertebral body fixation, especially in cases with severe vertebral body collapse, marked translational instability, or substantial torsional instability [10].

Stainless steel wire has several practical advantages as a binding material, including rigidity, radiographic visibility, and the ability to generate strong fixation by twisting. However, these same features may also be disadvantageous in small spinal constructs. Because stainless steel wire is relatively stiff, force may be transferred over a limited contact area at the bone-hole margin, potentially increasing localized stress at the spinous process. This raises concern regarding bone-hole cut-through or spinous process fracture, particularly in small animals with thin and fragile spinous processes.

FiberWire (Arthrex, Naples, FL, USA) is a non-metallic braided suture composed of an ultra-high molecular weight polyethylene core with a braided polyester jacket. It has been used as an alternative to metallic wire in selected orthopedic tension band and cerclage applications [11,12,13,14]. Compared with stainless steel wire, FiberWire is more flexible and can be tied using knots. These characteristics may reduce irritation associated with sharp metal ends and may alter how loads are transferred between the binding material, K-wire staple, and spinous processes. However, FiberWire may also be associated with knot-security concerns, cyclic elongation, creep, radiographic invisibility, and progressive loosening over time [14,15].

Accordingly, the use of FiberWire in a spinal stapling construct represents not simply a change in material strength, but a transition from a relatively rigid metallic binding material to a more compliant non-metallic material. The mechanical behavior of the construct may therefore depend on both the local bone-hole interface and the response of the complete multi-segment construct.

The objective of this study was to compare the preliminary biomechanical behavior of FiberWire and stainless steel wire as binding materials in a K-wire spinal stapling construct using intact ex vivo rabbit thoracolumbar models. We hypothesized that FiberWire would provide gross motion restriction comparable to stainless steel wire, while differences in material compliance would produce distinct mechanical behavior at the isolated bone-hole interface and complete-construct levels.

## 2. Materials and Methods

### 2.1. Specimen Preparation

Twenty male New Zealand White rabbit cadavers were used. The cadavers had been euthanized for reasons unrelated to this study. Median body weight was 3.16 kg (range, 2.5–4.2 kg), and median age was 6.0 months (range, 4.8–7.0 months). These animals had been utilized as healthy controls with no history of skeletal disease or gross bone abnormalities. The use of previously harvested cadaveric tissues was exempt from additional institutional animal care and use committee review in accordance with institutional policy.

The skin and dorsal thoracolumbar fascia were incised near the midline. Thoracolumbar vertebral segments were dissected from the surrounding epaxial musculature. The multifidus, longissimus, and iliocostalis muscles were removed. L5 and L6 vertebrae were harvested for isolated bone-hole interface tensile testing, while T12–L4 specimens were prepared for full spinal stapling construct testing. Surrounding soft tissue was removed except for the supraspinous and interspinous ligaments. Specimens were wrapped in saline-soaked gauze, sealed in plastic bags, stored at −20 °C, and thawed at room temperature on the day before biomechanical testing. Only vertebrae that remained structurally intact during dissection and specimen preparation were included in the biomechanical tests. Specimens that sustained gross fracture during preparation, ROM assessment, or K-wire insertion were excluded from the corresponding analysis.

### 2.2. Bone-Hole Interface Tensile Test

To first evaluate the baseline micro-environment failure behavior of the individual attachment points, a separate tensile test was performed on the isolated spinous process bone-hole interface using the L5 and L6 specimens (Figure 1A). A 1.0 mm K-wire was used to create a bone hole at the base of the dorsal spinous process. The vertebral body was embedded in resin and fixed in a testing jig. Either No. 2 FiberWire or 0.76 mm stainless steel wire was passed through the bone hole. The two free ends of each material were individually gripped and pulled in tension without knotting or twisting. Tensile loading was applied at 10 mm/min using a universal testing machine (Instron 5569, Instron, Norwood, MA, USA). Maximum load was defined as the highest load recorded before failure at the bone-hole interface, spinous process fracture, or material-related failure.

### 2.3. K-Wire Spinal Stapling Construct Assembly

Following the isolated interface tests, the macro-construct performance was evaluated using the T12–L4 specimens. Bone holes were created at the base of the spinous processes of T13, L1, L2, and L3 using a 1.0 mm K-wire. A 1.0 mm K-wire was passed through the L3 bone hole and bent into a U-shape, positioning it along both sides of the spinous processes from T13 to L2 to function as a staple-like dorsal implant. The K-wire staple was then secured to the remaining bone holes using either No. 2 FiberWire or 0.76 mm stainless steel wire (Figure 2). The terminal vertebrae were individually embedded in putty within rectangular steel prisms for subsequent mechanical evaluation. During potting, the specimens were aligned according to the orientation of the spinous processes.

### 2.4. Gross Range of Motion (ROM) Measurement

Gross flexion–extension and lateral ROM were measured in intact controls (without implantation) and both construct groups. For flexion–extension ROM, each specimen was manually bent until firm resistance was reached, and the angle was measured using a mechanical goniometer centered at the L1–L2 intervertebral disc level. Left and right lateral bending were measured in the same manner. Specimen orientation during mechanical testing was standardized using the spinous processes and the flat surfaces of the rectangular steel prisms as reference landmarks. Ventral and lateral bending were performed in orthogonal planes, with the loading direction oriented parallel and perpendicular to the spinous processes, respectively. This measurement was intended to confirm gross motion restriction by the construct and was not designed to quantify segmental stiffness or neutral zone. All ROM measurements were performed once by a single investigator; intra-observer consistency was not assessed.

### 2.5. Three-Point Bending Test

Destructive three-point bending tests were performed using the universal testing machine under either ventral or right lateral bending at a cross-head speed of 50 mm/min (Figure 1B,C). Load was recorded continuously using Bluehill 3 software (Instron, Norwood, MA, USA) with a 1 kN load cell. Maximum load was defined as the highest load recorded before specimen failure or construct-associated failure.

### 2.6. Statistical Analysis

Statistical analysis was performed using SPSS software (version 29, IBM SPSS Inc., Chicago, IL, USA). Data are expressed as median and range (minimum, maximum). A *p* value < 0.05 was considered significant. ROM differences among control and construct groups were assessed using the Kruskal–Wallis test followed by Mann–Whitney post hoc tests with Bonferroni correction. Maximum loads in the bone-hole interface tensile testing and three-point bending tests were compared using the Mann–Whitney U test.

## 3. Results

### 3.1. Bone-Hole Interface Tensile Test

In the isolated bone-hole interface tensile test using the fifth and sixth lumbar vertebrae, the median maximum load was 89.0 N (range, 70.1–161.0 N) in the FiberWire group and 118.2 N (range, 69.6–194.7 N) in the stainless steel wire group. The difference between the groups was not statistically significant (*p* = 0.20; Figure 3). During destructive loading, failure was localized to the spinous process bone-hole margin and was characterized by bone cut-through, shearing, or microfracture of the tunnel margin rather than obvious rupture of the binding material.

### 3.2. Gross Range of Motion

Both the FiberWire and stainless steel wire constructs significantly reduced gross ROM compared with the intact control specimens (Table 1, *p* < 0.01). The median flexion–extension ROM decreased from 48.15° (range, 34.95–51.60°) in the intact control group to 14.20° (range, 8.85–15.30°) in the FiberWire group, corresponding to a 70.5% reduction, and to 12.00° (range, 10.70–15.50°) in the stainless steel wire group, corresponding to a 75.1% reduction. Similarly, the median lateral ROM decreased from 55.29° (range, 42.10–66.20°) in the intact control group to 11.20° (range, 9.20–14.20°) in the FiberWire group, corresponding to a 79.7% reduction, and to 16.85° (range, 12.85–18.90°) in the stainless steel wire group, corresponding to a 69.5% reduction. No statistically significant differences in gross ROM were detected between the FiberWire and stainless steel wire constructs in either plane.

### 3.3. Three-Point Bending Test

Under ventral bending, the median maximum load was 77.4 N (range, 65.6–93.8 N) in the FiberWire group and 50.0 N (range, 40.9–55.2 N) in the stainless steel wire group. The median maximum load was significantly higher in the FiberWire group (*p* < 0.05; Figure 4), representing a 54.8% higher value than that observed in the stainless steel wire group. Under lateral bending, the median maximum load was 14.6 N (range, 12.4–24.8 N) in the FiberWire group and 14.4 N (range, 12.6–16.5 N) in the stainless steel wire group. No statistically significant difference was detected between the two groups (*p* > 0.05).

## 4. Discussion

This study compared FiberWire and stainless steel wire as binding materials in a K-wire spinal stapling construct [10,16]. The relative mechanical behavior of the two materials depended on the testing configuration. Stainless steel wire showed a numerically higher median maximum load at the isolated bone-hole interface, although the difference was not statistically significant, whereas FiberWire produced a significantly higher maximum load in the complete construct under ventral bending. Both materials significantly restricted gross flexion–extension and lateral range of motion compared with the intact condition, and no statistically significant difference was detected between the binding materials.

In the isolated bone-hole interface tensile test, failure occurred at the spinous process bone-hole margin rather than through rupture of either binding material. The measured maximum load was therefore likely governed primarily by the local bone–material interface and the structural characteristics of the spinous process rather than by the intrinsic tensile strength of the materials. Thus, the isolated test should be interpreted primarily as an assessment of local attachment-site failure behavior rather than as a direct comparison of the ultimate tensile capacities of FiberWire and stainless steel wire. Stainless steel wire is a relatively stiff metallic monofilament, whereas FiberWire is a more compliant braided suture, and previous studies have demonstrated differences in stiffness and deformation behavior between these materials [14,17]. The numerically lower maximum load in the FiberWire group may have been influenced by local sliding or settling of the braided suture against the tunnel margin, potentially contributing to bone cut-through or a Gigli saw-like effect [18]. However, local suture motion and bone-hole deformation were not measured.

The relationship differed in the complete construct under ventral bending. In this loading direction, resistance is likely influenced substantially by the binding materials connecting the K-wire staple to the individual spinous processes. The contrast between the isolated bone-hole and complete-construct results also indicates that the mechanical response observed at a single attachment site may not fully predict the behavior of a multi-segment fixation system. Whereas the isolated test primarily reflected local failure at one bone–material interface, the complete construct incorporated several attachment points together with the K-wire staple. Previous biomechanical studies have demonstrated differences in stiffness and deformation behavior between stainless steel wire and FiberWire constructs [13,14,17,19]. One possible hypothesis is that the greater stiffness of twisted stainless steel wire may have permitted less local deformation and concentrated load at fewer bone-hole margins, whereas the greater compliance of FiberWire may have allowed gradual deformation among multiple attachment points. Related biomechanical studies have shown that spinal tension band and semi-rigid construct configuration can influence bending behavior and load transmission [20,21]. In multilevel spinal tethering, tether configuration can alter the forces carried by adjacent spinous-process attachment levels, supporting the general concept that load distribution can vary among multiple fixation points [22]. This may represent one possible explanation for the higher ventral bending maximum load of the FiberWire construct. Nevertheless, the present study did not directly measure strain distribution, bone-hole deformation, FiberWire elongation, knot behavior or K-wire micromotion; therefore, improved load sharing and bone protection cannot be regarded as proven mechanisms [23].

The absence of a material-related difference under lateral bending may reflect the structural arrangement of the K-wire staple. During lateral bending, the two limbs of the U-shaped K-wire extend longitudinally along both sides of several spinous processes and may act as a multi-segment dorsal splint. Because both longitudinal limbs span several adjacent vertebral levels, movement at an individual segment may also be mechanically constrained by the K-wire extending across neighboring spinous processes. Lateral resistance may therefore depend more strongly on the bending stiffness and geometric arrangement of the K-wire than on the specific binding material used to secure it. This interpretation is consistent with the ROM findings, because both constructs markedly reduced lateral ROM compared with intact controls despite showing no significant difference between binding materials. Thus, the lateral results should not be interpreted as an absence of stabilization, but rather as evidence that changing the binding material did not significantly alter construct behavior under the tested lateral loading condition. The dominant role of the K-wire in lateral bending remains a biomechanically plausible interpretation rather than a directly measured finding.

Replacing stainless steel wire with FiberWire also has practical implications. Stainless steel wire provides high stiffness, radiographic visibility, and fixation by twisting. Its radiopacity may facilitate assessment of implant position, although projection and implant location influence radiographic interpretation [24]. However, metallic wire can produce sharp cut ends and may undergo fatigue or failure at repeatedly deformed regions [13]. FiberWire avoids sharp metallic ends and may reduce metal-related irritation and imaging artifacts, but it is radiographically invisible, more expensive, and dependent on knot security. Creep, cyclic elongation, knot slippage, and progressive loosening are additional concerns [14,15]. These factors were not evaluated in this static ex vivo study.

From a biomechanical perspective, maximum load during destructive testing represents only one aspect of construct performance [23]. In the present study, gross motion restriction and destructive failure resistance were assessed separately and should therefore be interpreted together. Although FiberWire showed a higher maximum load under ventral bending, this finding should not by itself be interpreted as evidence of superior clinical stabilization. Both binding materials produced substantial ROM restriction, with no significant difference detected between the two constructs.

The clinical relevance of these findings is limited specifically to the use of FiberWire as a binding material within the tested K-wire spinal stapling construct [10]. The present study evaluated whether changing the material used to secure the dorsal K-wire altered construct behavior; it did not evaluate FiberWire as an independent spinal fixation method. In addition, neither binding material directly stabilized the vertebral body. The results should therefore not be extrapolated to FiberWire as a primary fixation material for unstable vertebral fractures and luxations, severe vertebral body collapse, marked translational instability, or torsional instability [7,25,26]. Such conditions may require techniques that directly engage the vertebral body or stabilize multiple vertebral columns [8,9,27,28].

### Limitations

This study has several limitations. Rabbit vertebrae differ from canine and feline vertebrae in morphology, bone density, spinous process dimensions, and mechanical flexibility, although rabbit spinal models have been used for preliminary biomechanical and comparative skeletal studies [29,30]. These species-related differences may influence the absolute mechanical response of the construct and bone-material interfaces, limiting direct extrapolation of the measured values to canine or feline patients. The use of purpose-bred rabbits permitted more consistent specimen selection with respect to age, body size, and skeletal condition than would generally be feasible with clinically sourced canine or feline cadavers, providing a relatively uniform preliminary biomechanical model for the present study. A fracture-luxation model was not created, and the study evaluated an intact multi-segment construct rather than restoration of stability in a destabilized spine. Accordingly, these findings should be interpreted as preliminary biomechanical observations in intact spines rather than as evidence of clinical performance in unstable vertebral fractures or luxations. Gross ROM was measured manually without a standardized bending moment and should not be interpreted as segmental stiffness or neutral zone. Load–displacement curves, objective stiffness, energy to failure, cyclic durability, creep, torsion, and axial compression were not assessed [23]. In clinical use, however, spinal fixation constructs are exposed to repeated loading rather than a single destructive event. Under repeated loading, material creep, knot settling, cyclic elongation, and progressive loosening may alter construct behavior even when immediate static strength is maintained. Knot security and radiographic assessment were also not directly evaluated in the present study. Therefore, the present findings are limited to the immediate static behavior of the constructs, and the higher ventral bending maximum load observed with FiberWire should not be interpreted as evidence of superior long-term durability. FiberWire and stainless steel wire were secured by knot tying and twisting, respectively, so the comparison reflects clinically applied fixation systems rather than intrinsic material properties alone. Although 20 rabbit cadavers were initially used, not all harvested specimens could be included in every analysis because only structurally intact vertebrae were considered eligible. Specimens that sustained gross fracture during preparation, ROM assessment, or K-wire insertion were excluded from the corresponding analysis. Consequently, the final group sizes were relatively small and differed among the biomechanical tests. No a priori power analysis was performed; therefore, the study was not prospectively sized to detect a predefined between-group effect. The relatively wide ranges observed in some outcomes, particularly the isolated bone-hole maximum loads, indicate considerable inter-specimen variability. In the presence of such variability and limited sample sizes, the precision of the estimated between-group differences is reduced, and modest material-related effects may remain undetected. This is particularly relevant to lateral ROM, in which a numerical difference was observed between the FiberWire and stainless steel wire groups despite the absence of statistical significance. Accordingly, a type II error cannot be excluded, and the non-significant findings should not be interpreted as evidence of equivalence between the two binding materials.

## 5. Conclusions

FiberWire provided gross flexion–extension and lateral motion restriction comparable to stainless steel wire in a K-wire spinal stapling construct. FiberWire demonstrated a higher maximum load under ventral bending, whereas no significant advantage was found at the isolated bone-hole interface or under lateral bending. These direction-dependent results may reflect the predominant contribution of the binding materials during ventral bending and of the longitudinal K-wire limbs during lateral bending. Because these mechanisms were not directly measured, they should be regarded as plausible interpretations. FiberWire may be considered a non-metallic alternative binding material for dorsal K-wire spinal stapling, but superior overall stabilization, reduced bone-hole failure, and improved cyclic durability have not been established.

## Figures and Tables

**Figure 1:**
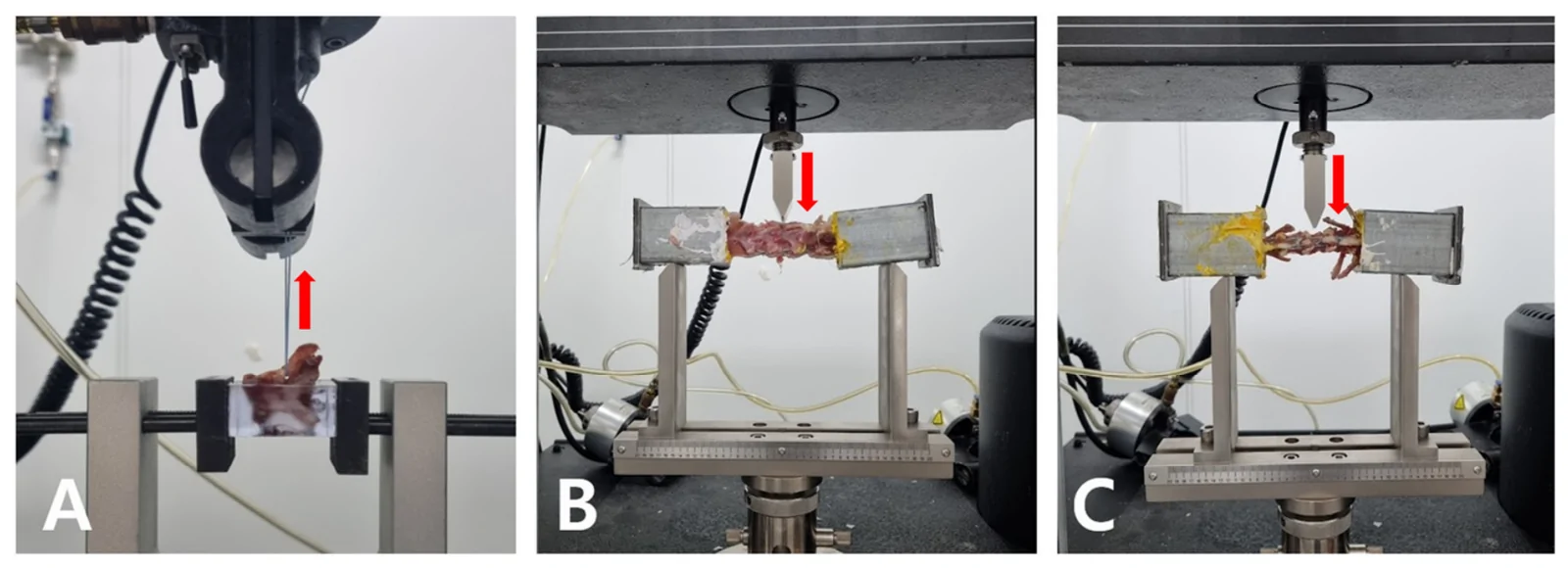
Biomechanical testing configurations using a universal testing machine. (**A**) Isolated bone-hole interface tensile test. (**B**) Three-point bending test under ventral loading. (**C**) Three-point bending test under lateral loading. Red arrows indicate the direction of applied loading. Maximum load was defined as the highest load recorded before specimen or construct-associated failure.

**Figure 2:**
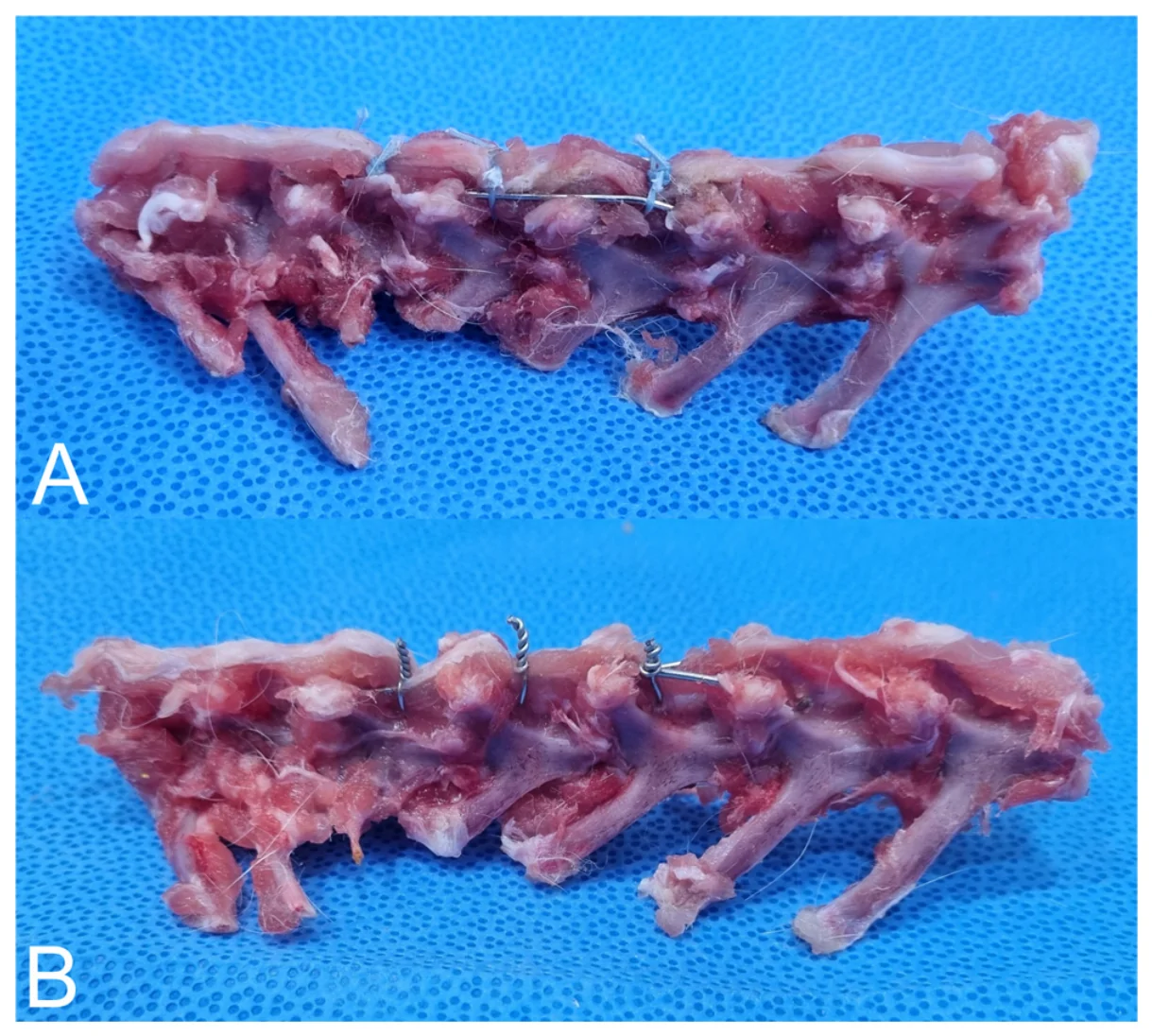
Kirschner wire spinal stapling constructs. (**A**) Construct secured with No. 2 FiberWire. (**B**) Construct secured with 0.76 mm stainless steel wire. In both panels, the cranial (thoracic) side is positioned to the left and the caudal (lumbar) side to the right.

**Figure 3:**
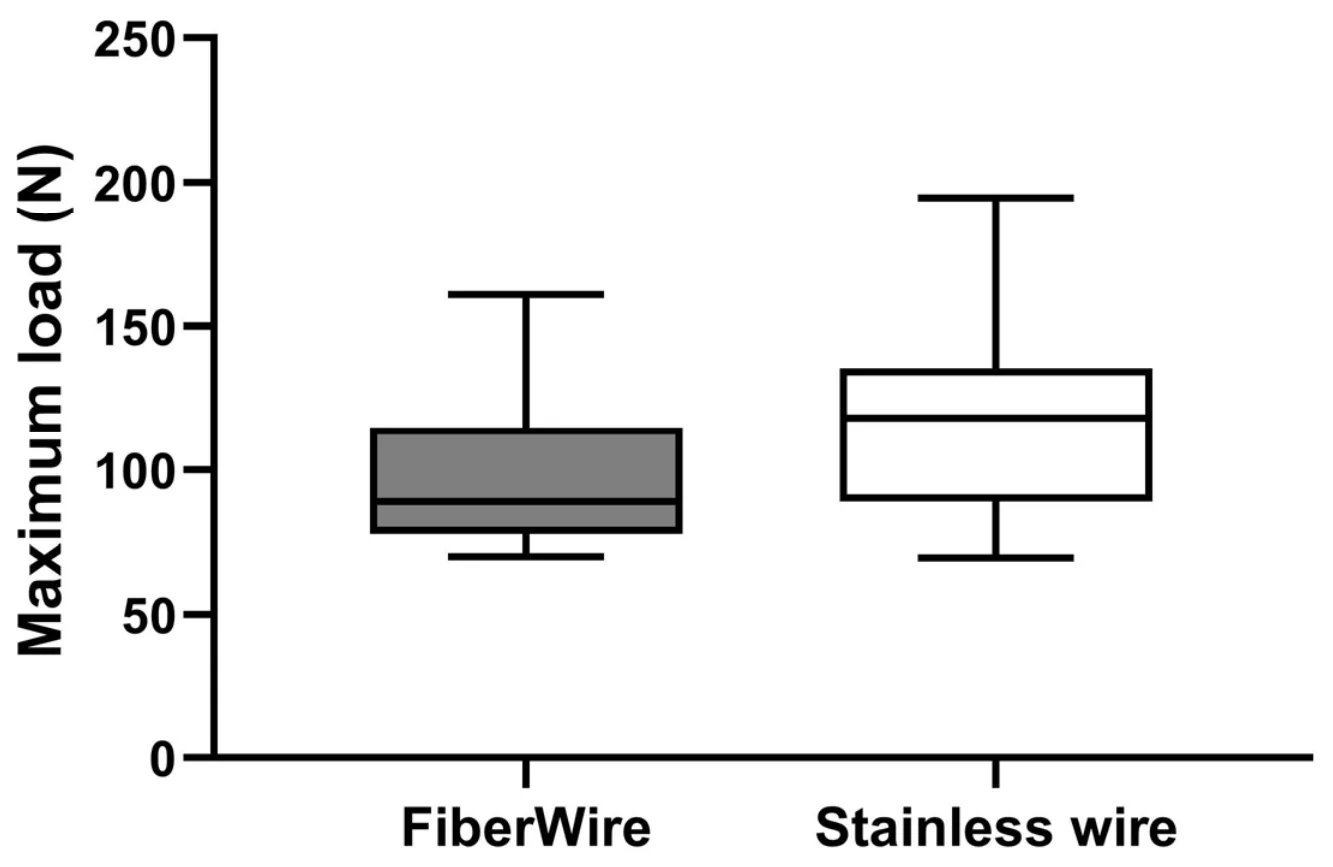
Comparison of failure strength at the isolated spinous process bone-hole interface. Maximum loads of the FiberWire and stainless steel wire groups during the isolated bone-hole interface tensile test utilizing the fifth and sixth lumbar vertebrae. Maximum load values are expressed in Newtons (N). No statistically significant difference in structural failure strength is observed between the two binding materials (*p* > 0.05). The FiberWire group represents isolated tensile test specimens utilizing No. 2 FiberWire (*n* = 14), and the stainless steel wire group represents specimens utilizing 0.76 mm stainless steel wire (*n* = 15).

**Figure 4:**
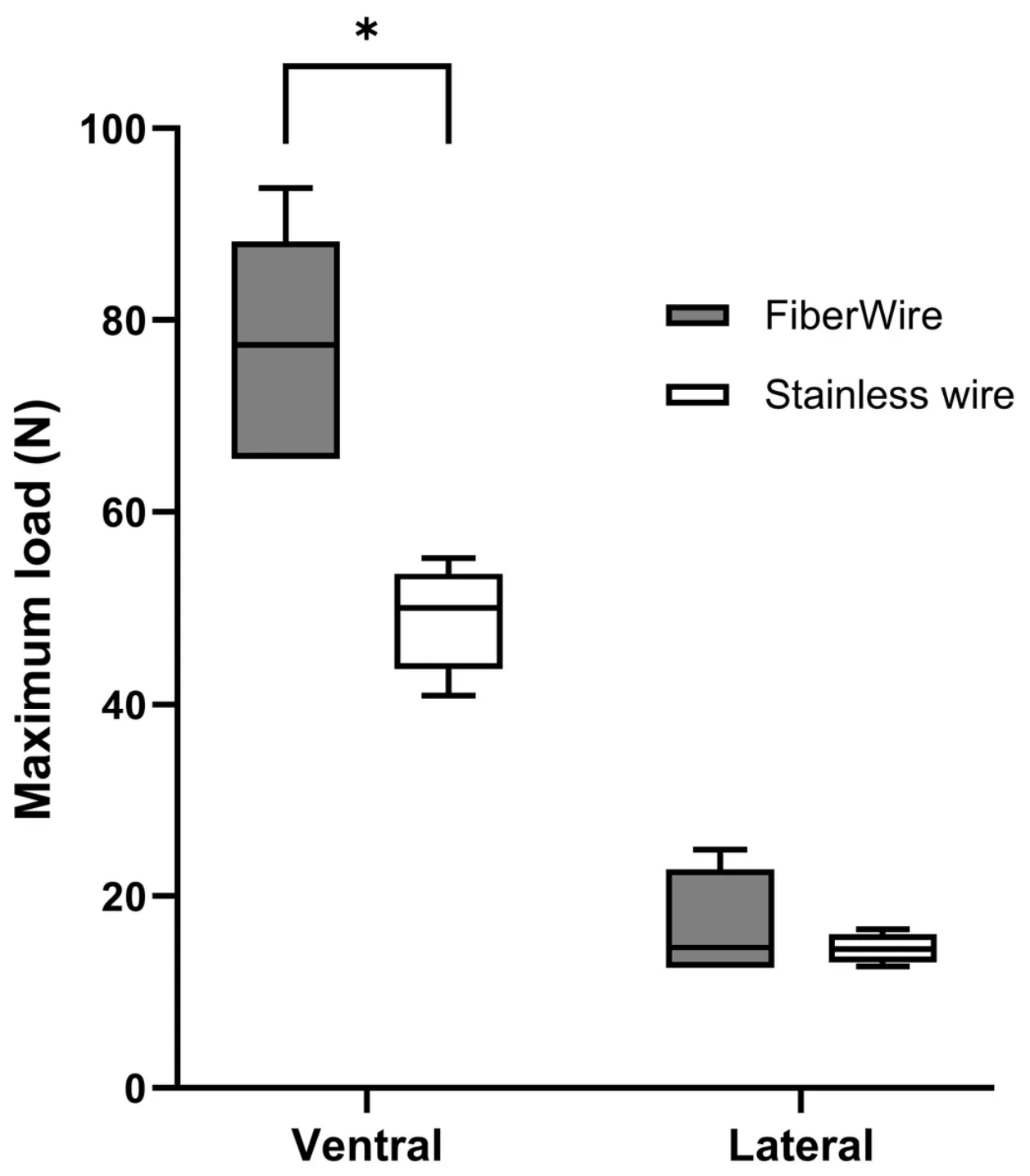
Structural failure strength of the multi-segment spinal stapling constructs under destructive three-point bending. Maximum loads of the FiberWire and stainless steel wire construct groups during the destructive three-point bending test under different loading directions. Under ventral bending, the FiberWire construct group exhibits a significantly higher median maximum load compared with the stainless steel wire construct group (*, *p* < 0.05). Conversely, under lateral bending, no statistically significant difference in structural resistance is observed between the two binding materials (*p* > 0.05). The ventral bending groups include specimens evaluated in the ventrodorsal direction utilizing either FiberWire (*n* = 5) or 0.76 mm stainless steel wire (*n* = 5). The lateral bending groups include specimens evaluated in the lateral direction utilizing either FiberWire (*n* = 4) or 0.76 mm stainless steel wire (*n* = 4).

**Table 1:** Comparison of ROM measurements among the control, FiberWire and stainless steel wire.

ROM (°)	Control (*n* = 5)	FiberWire (*n* = 7)	Stainless Steel Wire (*n* = 5)
Flexion–Extension	48.15 (34.95, 51.60)	14.20 * (8.85, 15.30)	12.00 * (10.70, 15.50)
Reduction (%)		70.5	75.1
Lateral	55.29 (42.10, 66.20)	11.20 * (9.20, 14.20)	16.85 * (12.85, 18.90)
Reduction (%)		79.7	69.5

Data are median (minimum, maximum). Percentage reductions were calculated from group median ROM values relative to the control median. * *p* < 0.01 compared with control. No significant difference was detected between FiberWire and stainless steel wire. ROM, range of motion.

## Data Availability

The raw data supporting the conclusions of this article will be made available by the authors on request.

## Abbreviations

The following abbreviations are used in this manuscript:

K-wire	Kirschner wire
ROM	Range of motion
